# Coral snakes predict the evolution of mimicry across New World snakes

**DOI:** 10.1038/ncomms11484

**Published:** 2016-05-05

**Authors:** Alison R. Davis Rabosky, Christian L. Cox, Daniel L. Rabosky, Pascal O. Title, Iris A. Holmes, Anat Feldman, Jimmy A. McGuire

**Affiliations:** 1 Department of Ecology and Evolutionary Biology and Museum of Zoology, University of Michigan, 1109 Geddes Avenue, Ann Arbor, Michigan 48109, USA; 2 Museum of Vertebrate Zoology and Department of Integrative Biology, University of California, Berkeley, 3101 Valley Life Sciences, Berkeley, California 94720, USA; 3 Department of Biology, Georgia Southern University, PO Box 8042, Statesboro, Georgia 30460, USA; 4 Department of Biology, The University of Texas, Arlington, Texas 76019, USA; 5 Department of Zoology, Tel Aviv University, Tel Aviv 69978, Israel

## Abstract

Batesian mimicry, in which harmless species (mimics) deter predators by deceitfully imitating the warning signals of noxious species (models), generates striking cases of phenotypic convergence that are classic examples of evolution by natural selection. However, mimicry of venomous coral snakes has remained controversial because of unresolved conflict between the predictions of mimicry theory and empirical patterns in the distribution and abundance of snakes. Here we integrate distributional, phenotypic and phylogenetic data across all New World snake species to demonstrate that shifts to mimetic coloration in nonvenomous snakes are highly correlated with coral snakes in both space and time, providing overwhelming support for Batesian mimicry. We also find that bidirectional transitions between mimetic and cryptic coloration are unexpectedly frequent over both long- and short-time scales, challenging traditional views of mimicry as a stable evolutionary ‘end point' and suggesting that insect and snake mimicry may have different evolutionary dynamics.

## Abstract (web summary)

Toxic and venomous species often have conspicuous warning colouration that is mimicked by harmless species. Here, Davis Rabosky *et al*. combine phylogenetic and biogeographic analyses to reveal that mimicry of venomous coral snakes has been a major driver of snake colour evolution in the New World.

## Graphical abstract

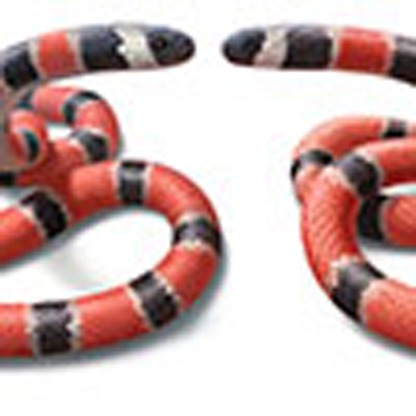

In the first formalization of mimicry theory in the 1860s (ref. 1), brightly banded neotropical snakes were presented as the flagship example of ‘true mimicry' in vertebrates. Similar to well-known examples of toxic butterfly^2^ and wasp^3^ mimicry, the conspicuous red–black banded (RBB) colour pattern in some harmless snakes is thought to arise through mimicry of highly venomous coral snakes throughout North and South America^4^. This striking convergence between distantly related species has motivated a century of experimental research demonstrating that predators innately avoid coral snake-like colour patterns and that this avoidance provides a selective advantage to mimetic coloration over other colour patterns^5^,^6^,^7^,^8^.

However, many researchers still question the extent, or even existence, of coral snake mimicry because snakes clearly violate two major theoretical predictions that should hold true for mimicry to persist over long periods of time^1^,^9^,^10^: (1) that mimics should not occur outside the geographic range of the model species, and (2) that models should be more abundant than mimics, or at least near a ratio of 1:1 (refs 11, 12, 13, 14, 15). In fact, snakes include the only known example of a Batesian mimic that occurs entirely outside the geographic range of any model species (the California Mountain Kingsnake, *Lampropeltis zonata*^16^). Although there is empirical evidence in insect mimicry that model abundances can be lower than mimics^17^,^18^ and a few species are found outside the range of their models^19^, insects generally conform to these theoretical predictions. Typically, model-mimic pairs show only narrow zones of mismatch in geographic distribution of coloration^18^,^20^,^21^,^22^,^23^ and models are often more common than mimics^24^. As both taxa share birds as a key predator class, the failure to meet these predictions in coral snakes and their mimics remains a puzzling evolutionary paradox.

Current theoretical models, however, ignore the contribution of both (a) large-scale biogeographic patterns of species diversity and (b) the evolutionary history of the mimetic phenotype to the current distribution of species with conspicuous colour patterns. Although these effects are presumed to be minimal relative to the rate of natural selection within populations, they have not been well tested because most species occurrence records remain non-digitized and precise taxonomic relationships unknown, especially for insects. Because species are distributed non-randomly in space^25^ and phenotypic traits often show phylogenetic signal^26^, convincing tests of mimicry should incorporate biogeographic data in an evolutionary framework^3^,^27^,^28^, especially across large clades.

Here, we leverage large-scale digitization of vertebrate museum records with advances in our understanding of the phylogenetic relationships among squamates^29^ to reconcile the conflict between theoretical predictions and empirical observations, and provide a comprehensive test of mimicry in space and time. We created geographic range polygons for over 1,000 New World snake species from both published records and occurrence data (*n*=299,376 specimens) to test for a positive spatial correlation between coral snakes and their presumed mimics while controlling for geographic variation in species richness. Next, we used a phylogenetic framework to reconstruct origins of mimicry and rates of colour evolution across snakes at multiple time scales to characterize transitions between cryptic and conspicuous coloration, and test for temporal correlations with coral snake co-occurrence. We find widespread evidence that coral snakes drive the distribution of harmless RBB snakes over the entire Western Hemisphere and through 40 million years of snake evolution. However, we also demonstrate striking lability of coloration that challenges traditional ideas about the evolutionary stability of mimicry systems and identifies new research targets for understanding phenotypic convergence.

## Results

### Spatial tests of co-occurrence between models and mimics

**Figure 1:**
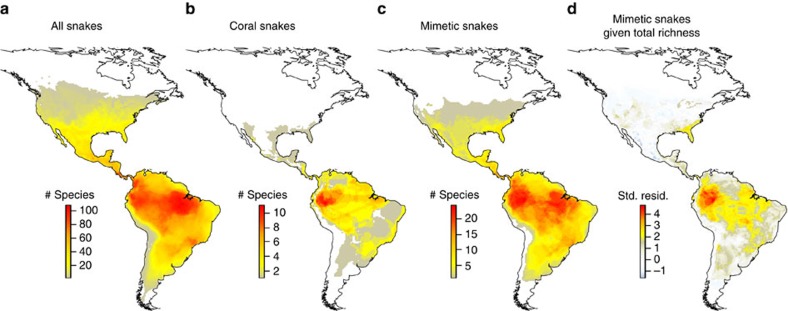
Spatial correlation between coral snakes and their mimics. (**a**) Snakes (*n*=1,081 species) are distributed non-randomly through the New World, with the centre of diversity in the western Amazon Basin of South America. (**b**) Spatial distribution of venomous coral snakes (Elapidae; *n*=78 species). (**c**) Spatial distribution of harmless mimetic snakes (Colubridae; *n*=133 species). (**d**) Residual mimetic species richness after removing the effect of overall species richness shows a strong positive spatial correlation to coral snake richness patterns in **b**.

**Figure 2:**
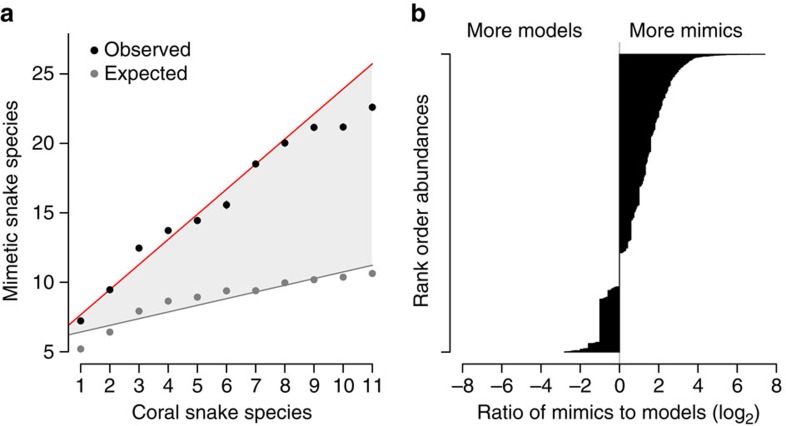
Species richness and abundance relationships favour mimics over models. (**a**) After accounting for the positive relationship between mimetic and overall species richness (expected values, grey), coral snakes have a linear, additive effect such that ∼2 more mimetic species can be supported for every additional coral snake species. (**b**) Across the western hemisphere, abundance ratios of coral snakes to their colubrid mimics suggest that mimetic snakes are far more numerically abundant than their co-occurring models.

Contrary to historical arguments citing widespread evidence of critical violations to mimicry theory in snakes, we found that predictions of co-occurrence between models and mimics are well supported once spatial and phylogenetic non-independence are taken into account. After controlling for geographic patterns of overall species richness (Fig. 1a), we found that coral snakes are a strong predictor of the number of mimetic species (Supplementary Data 1) using both spatial autoregressive (SAR) models (*Z*_coral_=2.081, *P*=0.037; *Z*_full_=22.244, *P*<0.001; Supplementary Fig. 1) and permutation tests that randomized species identities (*P<*0.001; Fig. 1 and Supplementary Fig. 2). We found that areas of high coral snake richness contain an extreme excess of mimics relative to expectations based on overall species richness alone (Fig. 1d). This positive correlation between models and mimics appears to be a general feature of all biomes south of the boreal zone^30^, indicating that it is not restricted to a single major habitat. Even within widespread lowland tropical forest, peak residual mimetic richness is restricted to a small fraction of total forest area, specifically coincident with peak coral snake richness (compare Fig. 1b–d). This coral snake effect corresponds to a 50–100% increase in mimetic richness over expected values from null distributions (Fig. 2a, Supplementary Fig. 3). However, we also found that the ‘mimetic excess' problem^12^ is even larger than historically suggested, with 2–6 times more mimetic than model species present in a given locality (Fig. 2a, Supplementary Data 2 and 3). We see the same pattern in individual snake abundance data, such that 70% of grid cells where coral snakes occur have abundance ratios biased towards mimics rather than models, often to great excess (Fig. 2b).

### Phylogenetic analyses of colour pattern evolution

**Figure 3:**
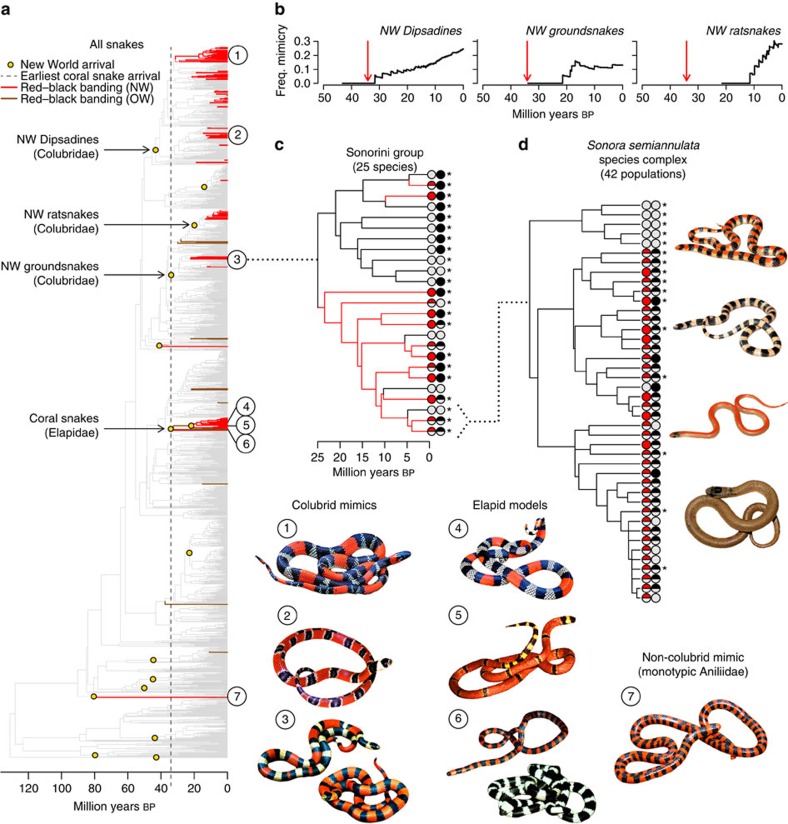
Repeated evolutionary transitions among colour patterns in snakes. (**a**) Ancestral state reconstructions show that RBB colouration has evolved independently at least 26 times across snakes, including six times outside of New World (NW) colubrids and Elapid coral snakes. Numbers denote phylogenetic placement of corresponding snake images. Species 3 and 6 have sympatric colour polymorphism. (**b**) Colubrid mimicry arose after both arrival in the NW (oldest black points) and sympatry with coral snakes (red arrow), with increasing accumulation of mimetic lineages over time. (**c**) Addition of missing taxa to a single clade recovers greater colour variation and faster phenotypic evolution. Tip state symbols show presence and absence of the red and black colour pattern components, with bicoloured points representing colour polymorphism (grey for absence). Asterisks denote co-occurrence with coral snakes. (**d**) Trait mapping of intraspecific colour variation shows that polymorphism is the most common state and that RBB coloration does not depend on sympatry with coral snakes. Righthand images show four sympatric colour morphs found in populations with RBB polymorphism. The species names, photo credits (used with permission), and field collector/tissue numbers (when available) for the images in **a** are as follows: (1) *Oxyrhopus trigeminus*, Laurie J. Vitt, LJV-17825; (2) *Erythrolamprus mimus*, Edmund D. Brodie III; (3) *Sonora mutabilis*, Thomas J. Devitt, JAC-23362 and JAC-23363; (4) *Micrurus brasiliensis*, Donald B. Shepard; (5) *Micrurus diastema*, Jonathan A. Campbell, JAC-23126; (6) *Micrurus multifasciatus*, Edmund D. Brodie III; (7) *Anilius scytale*, Donald B. Shepard. For **d** the *Sonora semiannulata* colour morphs have the following credits from top to bottom: (1) mimetic morph, Yann Surget-Groba, ADR-BAL1M; (2) banded morph, Yann Surget-Groba, ADR-HIL1B; (3) striped morph, Yann Surget-Groba, ADR-Tor1S; (4) uniform morph, Alison R. Davis Rabosky and Christian L. Cox, CLC-291.

Across all snakes, phylogenetic ancestral state reconstructions using maximum likelihood, Bayesian random clock models and parsimony approaches (Fig. 3a, Supplementary Figs 4–6) concordantly reveal at least 19 independent origins of mimetic coloration within colubrid snakes that are temporally correlated to co-occurrence with New World coral snakes, especially within the subfamily Dipsadinae. As predicted by Batesian mimicry theory, the origins of RBB coloration in New World colubrids postdate the arrival of coral snakes in all cases where the origin of the trait can be reliably inferred (Fig. 3b; Supplementary Note 1). We also found substantial rate heterogeneity among clades (Supplementary Fig. 7, Supplementary Data 4), such that New World Dipsadine snakes had a transition rate from a cryptic pattern to red–black banding that is five times the background rate across snakes worldwide.

### Geographic reconstruction of mimicry losses

**Figure 4:**
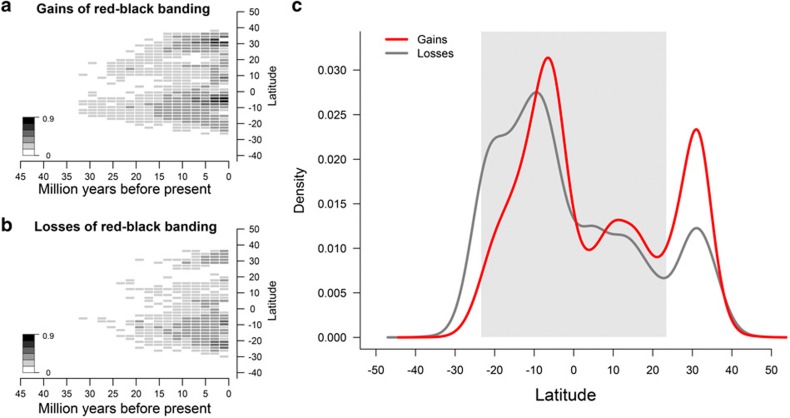
Geographic distribution of RBB gains and losses. Stochastic character mapping of (**a**) gains and (**b**) losses of RBB coloration co-modelled with geographic ranges by latitudinal midpoint show that colour transitions are most frequent in two distinct geographic areas (one tropical and one north temperate) and that most change has occurred over the past 15 million years. Colours of grid cells reflect the mean number of gains or losses per simulated character state distribution scaled to the maximum number of either transition so that gains and losses are directly comparable. (**c**) Marginal density plots of gains and losses with respect to latitude show that the majority of gains, but especially of losses, have occurred in the tropics. Grey shading denotes the boundaries of the tropics between 23° S and 23° N.

Most surprisingly, phenotypic rate analyses also suggest rapid bidirectional transitions among colour patterns that scale with time (Fig. 3; Supplementary Fig. 8). Losses of RBB coloration are numerous and scattered across the phylogeny, such that clades of Dipsadines that are broadly sympatric with coral snakes have more losses than allopatric Colubrine clades in North America (for example, *Lampropeltis*; Supplementary Figs 5 and 6). In a further characterization of these losses through phylogenetic reconstructions of ancestral lineage latitude, we found that the majority of both gains and losses occurred in the tropics (23° N—23° S), with most of the recovered changes happening over the last 15 million years (Fig. 4). Although the two temperate Colubrine clades with slower transition rates combine to produce noticeable RBB loss rates in the northern temperate (23—35° N) zone, we found that losses are disproportionately common in the tropics (Fig. 4c). The number of losses of RBB coloration in the tropics exceeds the number of losses in the northern temperate zone by a factor of 5.7, primarily due to the extreme transition rates in several subclades of the tropical Dipsadines, even though the tropics are the centre of coral snake diversity and the region inferred to have the longest presence of models given the taxa present in the phylogeny.

### Analyses of mimetic colour polymorphism

We then took one of the mimetic clades reconstructed with the slowest rate of phenotypic evolution (subfamily Colubrinae, tribe Sonorini) and reanalysed the history of mimicry after in-depth sampling of the species in the clade and populations within a species^31^ (Fig. 3c,d; Supplementary Fig. 9). We found a classic fractal pattern of colour diversity in which sequentially more variation and faster transitions are found as time scale decreases (compare Fig. 3a–d). We found that even though we had originally reconstructed this clade as a single, old (25 Mya), stable origin of mimicry, we recovered an additional two origins and three losses of RBB colouration by simply adding missing species to the tree (Fig. 3c). This finding represents a sixfold increase in the rate of phenotypic evolution, suggesting that the high rates we recovered across mimetic snakes may still be underestimates of true transition rates. These within-clade analyses also revealed rampant polymorphism in the two warning signal colour components (Fig. 3c,d), with half of the species in this mimetic clade (11/22 species) and 85% of populations within a single species complex (35/41 populations) displaying polymorphism for red and/or black banding (Supplementary Data 5). As we found that at least a third of all New World mimics and a fifth of all coral snakes are polymorphic for RBB signal components (Supplementary Data 1), the Sonorini group represents a common condition. We then repeated the spatial tests by examining the effect of coral snake richness on mimetic polymorphism, and we again found a positive correlation, disproportionately driven by areas with high coral snake richness (*Z*_coral_=2.783, *P*=0.005; *Z*_full_=48.364, *P*<0.001; permutation test non-significant, but same trend; Supplementary Figs 1–3).

## Discussion

Overall, we found strong support for the concordance of coral snakes and their mimics in both space and time, a key prediction of Batesian mimicry theory. However, our findings of (1) an excess of mimetic richness and abundance in both allopatry and sympatry with model species, and (2) rapid rates of transition among phenotypic states, with many reversals back to a cryptic colour pattern even in sympatry with coral snakes, show surprising contrast to theoretical predictions and empirical patterns found in many insect systems. First, when added to the mounting evidence from insects that model abundance does not have to be high for mimicry persist^17^,^18^, our empirical data suggest that mathematical models that constrain Batesian mimics to be rarer than their toxic models are at odds with the biological realities of mimicry, perhaps especially in cases of lethal model toxicity-like coral snakes. Early experimental evidence with starlings and quinine-laced mealworms^32^ suggested that Batesian mimics can derive benefit even when they outnumber models by a ratio of 9:1, and our data support this conclusion as well. Also of interest is the relationship between the number of mimetic species supported by areas of different coral snake richness where models and mimics are entirely sympatric (as in Fig. 2a). Whether mimetic and/or model richness can increase indefinitely, whether that relationship is equilibrial, and what similar patterns might look like in insects, are all unknown.

The most important result of this study is the evidence of widespread evolutionary losses of mimetic coloration, which we observed both at broad phylogenetic scales (Fig. 3a, Supplementary Figs 4–6) and within the *Sonora* complex (Fig. 3c,d). Such a pattern has never been found in insects (one loss reported in ref. 28, refuted in ref. 33), despite phylogenetic reconstruction of mimetic coloration in Batesian systems as taxonomically widespread as butterflies^22^,^33^,^34^, hoverflies^3^ and ant-mimicking spiders^20^,^35^. In fact, this lack of loss in insects has caused researchers to hypothesize that Batesian mimicry may be a one-way street, or directional trajectory, from which reversion to crypsis is not likely^34^,^36^. However, the sheer number and phylogenetic distribution of losses in both of our data sets strongly rejects the idea that mimicry is irreversible in snakes.

Although it is possible that mimicry itself is much more stable in insects, perhaps due to differences in historical range stability of the models, another explanation for these contrasting patterns of phenotypic evolution may be that mimetic phenotypes in snakes and insects are associated with differential extinction rates^34^. In butterflies, it has been proposed that quick extinction of conspicuous mimics when in allopatry to the model would eliminate the phylogenetic signature of non-mimics nested within clades of mimics^34^. In this case, the rate-limiting step on the evolutionary persistence of a reversal to crypsis might be how fast the mimetic signal can break down once mimics are in allopatry given the genomic architecture of mimicry loci^34^, which are often linked into supergenes^37^,^38^,^39^,^40^. In snakes, this scenario could be altered not only because of different genomic architecture, but also through reduced predation risk to allopatric mimics mediated by increased relative body size and lethality of venomous snakes over insects^41^. If allopatric snake mimics are less likely to go extinct than allopatric insect mimics because of a stronger generalized avoidance of coral snake coloration by predators, then these lineages are simply more likely to persist long enough to both lose mimetic coloration and be detected in phylogenies of extant taxa.

However, our surprising observation that most losses of RBB coloration occurred in the tropics, in clades broadly concordant with coral snakes, suggests that major latitudinal range shifts of models and subsequent allopatry of mimics may not be driving the bulk of mimicry losses in snakes (although model range shifts, especially due to historical glacial cycles, may explain losses recovered in the temperate zone). While our results do not preclude the possibility that tropical losses could be driven by shifting patterns of geographic overlap involving tightly matched model-mimic species pairs, generalized avoidance of coral snake colouration by tropical predators^5^,^6^ and the relatively continuous distribution of coral snakes in the tropics (Fig. 1b) suggest that this is not where we should find the highest frequency of loss if absence of models is the driving factor in mimicry breakdown. The nature of colour polymorphism—which and how many morphs are present, how polymorphism scales with model diversity and the phylogenetic distribution of polymorphic mimics—may be helpful in informing this debate. As the frequency-dependent nature of Batesian mimicry is expected to generate colour polymorphism^42^, colour variation can be leveraged to identify the factors that promote the most polymorphism. In snakes, polymorphism is higher than expected both where coral snake richness is high and where it is low (Supplementary Fig. 2e), suggesting a mixture of processes driving colour variation. A similar analysis in insects, especially highly polymorphic butterflies, would better inform how polymorphism might factor into the probability of complete loss of a mimetic phenotype.

Although the increased body size and lethality of venomous snakes over insects relative to their avian predators may be important^41^, both empirical and theoretical work is needed to determine whether these factors can truly explain such different dynamics and stability of mimicry systems across taxa^43^. However, too much of the variation in mimetic snakes is explained by coral snakes to argue that snake mimicry is implausible based on data such as empirical abundance parameters. We propose that future research is best served by a working hypothesis in which all New World RBB snake species are considered mimics until proven otherwise, which is philosophically more similar to investigative approaches in insect mimicry. With this perspective shift, we may finally understand how systems united by the same selective forces on the same class of predators can have such divergent dynamics through space and time.

## Methods

### Phenotypic classification

To create a list of mimics in the New World and RBB snakes in the Old World (Supplementary Data 1), we classified snake colouration following the phenotypic codes defined in ref. 44. For New World species, we included any species that had (1) uniform red coloration, usually with black accessory colouration such as a black nuchal collar (codes that start with U), (2) any variation of bicolour red and black crosswise banding (codes that start with B), (3) any variation of tricolour red, black and yellow/white banding (codes that start with T) or (4) any variation of quadricolour grey, red, black and yellow/white banding (codes that start with Q). Following ref. 44, the criteria for including a New World species in this list of mimetic species was (a) an emphasis on the presence of red pigment in either full dorsal coloration or in crosswise bands, and (b) including all colour patterns found in New World coral snakes. Thus, species with red dorsal or lateral striping in the absence of black bands (for example, *Thamnophis sirtalis*) or a pure black dorsum and red lateral/ventral coloration (for example, *Boiruna maculata*, *Pseudoboa martinsi*) were not included as mimics. For Old World species, we conservatively included only species with bicolour, tricolour or quadricolour banding that included both red and black pigmentation. Although we used refs 44 and 45 as the basis for our phenotypic scoring, we updated all species to current taxonomy and added both newly described species and Old World species that were not included in either original publication. We classified species as polymorphic if individuals had (a) discrete variation in presence/absence of red or black pigmentation, or (b) multiple major phenotype classes (for example, both bicolour and tricolour morphs). We assigned 255 snakes worldwide to these colouration categories (231 New World species, 24 Old World species), confirming that RBB colouration is a rare phenotype with viewed across all snakes (out of ∼3,500 described species, 7.3%). Note that we considered any species that did not have RBB coloration as ‘cryptic' for simplistic comparison, but we do not imply anything formal about conspicuity by using this designation or degree of mimicry by using ‘mimetic'.

### Geographic range construction

We constructed geographic ranges for 1,107 out of 1,328 (83.4%) continental species of New World snakes. Out of these species, 78 (out of 80 identified) were coral snake species, 133 (of 151) were mimetic species and 46 (of 50) were polymorphic mimics. We compiled geographic range data in three ways: (1) when available, using known species ranges downloaded from IUCN Red List of Threatened Species (www.iucnredlist.org, accessed on 1 February 2015), (2) hand compiling ranges from published sources or (3) acquiring point occurrence data (*n*=299,376 unique records) from the VertNET (www.vertnet.org) and GBIF (www.gbif.org, both accessed on 26 May 2015) data portals to assemble ranges *de novo*. To standardize taxonomy among sources, we matched species names to synonym listings scraped from the Reptile Database (reptile-database.reptarium.cz, accessed on 5 October 2015) using a two-step process that searched by date range (first 1950 to present, then 1800–1950 if there were no hits), multiple combinations of former genus and species synonyms, and allowed fuzzy character matching (*n*=2) to accommodate gender agreement changes. We also filtered occurrence data by the species-specific country listings of Reptile Database (country names standardized) and cross-referenced these listings with the global invasive species database (www.issg.org, accessed on 15 January 2015) so that we retained only occurrences from the species' native distributions. For all occurrences that were not georeferenced but did have text-based municipality information, we jittered occurrence points randomly within the political boundaries of that municipality (usually ‘state').

To construct species range polygons from occurrence data, we used a mixture of alpha hull, minimum convex hull and point buffering methods depending on the number of records for each species. For all species with five or more occurrences, we built alpha-hull polygons with the ‘alphahull' package^46^ in R v3.1.2, sequentially increasing the alpha parameter value until a contiguous species range was created that contained at least 99 per cent of the occurrence records. For species with 3–5 occurrences, we generated a minimum convex hull using the ‘rgeos' R package^47^. For species with 1–2 records, we buffered each point with a 0.5° radius. All species ranges were then clipped to the coastline using ‘rgeos' and the GSHHS coastlines data set^48^.

For all coral snakes and five mimics, we then replaced the ranges we had generated from occurrences, or species with no occurrences, with ranges that were hand compiled from published sources as part of the Global Assessment of Reptile Distributions (http://www.gardinitiative.org) international consortium^49^. Because coral snakes were the independent variable of interest in our statistical models, it was important to have the most accurate ranges possible and justified using time-intensive construction ‘by hand.' However, our results and overall conclusions were qualitatively similar even when analyses were restricted to only ranges reconstructed from occurrence data (thus excluding both IUCN and coral snake range replacements; permutation test for mimicry, *P*<0.001).

To tally species counts for subsequent spatial analyses, we created a raster of species richness for each species set (all snakes, coral snakes, mimetic snakes and polymorphic mimics) at two different latitude/longitude grid resolutions from the overlay of all assembled species ranges. First, we chose a 2° by 2° grid resolution for its balance between accurately capturing species richness patterns without overly zero-weighting the distributions for the dependent variables subsequently analysed, which is a consequence of smaller grid cell size (Supplementary Data 2). Next, we used a 0.5° by 0.5° resolution to reduce the influence of habitat-driven spatial heterogeneity and increase the probability that two species co-occurring in a cell were truly coexisting on a spatial scale more relevant to avian predators (0.5°≈55 km at the equator; Supplementary Data 3). Species were considered present in a cell if their range covered 50% or more of that cell, and we used each cell's midpoint as its geographic coordinate. After discarding cells not on the mainland, we parsed this data set into two subsets of grid cells containing (a) at least one mimetic species (*n*=520 cells at 2°, 8,656 cells at 0.5°), or (b) at least one coral snake species (*n*=324 cells at 2°, 5,609 cells at 0.5°), and we ran all spatial analyses on each subset. We conducted the latter test to reduce distributional skew towards low values, but the results were qualitatively the same in all analyses (Supplementary Fig. 3). We conducted analogous pruning for analyses of mimetic polymorphism (*n*=471 cells at 2°, 7,196 cells at 0.5°).

For analyses of relative abundances of coral snakes and their mimics, we used all the individual occurrence points and generated the same rasters above, but with individual specimen counts for all snakes, coral snakes and mimetic species (Supplementary Data 2 and 3). To compare abundances of models and mimics for each cell, we added 1 to each total before taking the log2 of the ratio of mimetic snake to coral snake individuals.

To test whether randomizing non-georeferenced points within states/provinces biased our conclusions, we also implemented a ‘conservative' approach in which we first reduced the occurrence data set for each species to include only its georeferenced records (*n*=211,614 records total; mean=40% of total records per species), constructed ranges as above, and designated that the ‘known range.' Then, we added each non-georeferenced record along the border of its collection state at the point with the shortest straight line distance to the known range before reconstructing a revised range polygon. As our overall conclusions do not change with this more conservative approach (Supplementary Figs 2c and 3d, this comparison suggests that our original municipality-guided randomization added minimal inappropriate bias to the data, but did work to smooth ranges, especially in Brazil, and thus functioned similarly to a standard interpolation algorithm. For a conservative approach to the abundance analyses, we repeated analyses using only georeferenced records and again found the same results (Supplementary Fig. 3l).

All of the above methods for scraping collections database aggregators, filtering records and using occurrence information to reconstruct species ranges have been generalized to accommodate a range of vertebrate taxa and are now available in the R package ‘rangeBuilder' downloadable from CRAN (https://cran.r-project.org).

### Spatial analysis

To test the spatial relationship between coral snakes and their mimics, we performed SAR error models using the ‘spdep' R package^50^. We tested for spatial autocorrelation using sequentially increasing neighbourhood sizes (stepwise by 50 km) beginning at the smallest size for which neighbours could be computed for every cell and compared model AIC scores (unweighted, weighted and inversely weighted by distance) to choose the best performing neighbourhood size and weighting regime for final analysis (Supplementary Fig. 1a–d). All models were multiple regressions of mimetic (or polymorphic) snake species richness on coral snake richness+total species richness. Although we found a significant interaction effect between coral snake and total species richness, it explained negligible variation and was removed (mimicry: adjusted *R*^2^=0.8992 for interaction model, adjusted *R*^2^=0.8980 for additive model; polymorphism: adjusted *R*^2^=0.3202 for interaction model, adjusted *R*^2^=0.3106 for additive model; full model *P*<0.001 in all cases). We computed Moran's I for each analysis to confirm model adequacy in removing spatial autocorrelation (Supplementary Fig. 1e–h). Note that due to the complexity of the neighbourhood matrices for 0.5° resolution analyses, we only ran SAR models at the 2° resolution.

To further account for complex spatial covariance effects (for example, continental shape) and distributional skew of diversity unable to be explicitly specified in SAR analysis, we also conducted a permutation test (*n*=1,000 iterations) to compare observed mimicry data with a null distribution that preserved spatial and species diversity structure. To generate null distributions, we randomized (without replacement) the species identity tags of all non-coral snake species range polygons before creating species richness rasters. Thus, each simulated raster had identical (a) range distributions, (b) species richness totals for each grid cell and (c) distribution of coral snakes as the observed data. However, the species name codes, which correspond to mimetic and polymorphic snakes, were randomized across the New World independently of coral snakes. We then compared linear regressions of both observed and simulated data to determine the increase of mimetic/polymorphic snake richness due to coral snake richness beyond the known positive effect of overall species richness and spatial autocorrelation. We calculated one-tailed *P* values as the number of simulated slopes equal to or greater than our observed slopes divided by the total number of simulations.

To calculate standardized residuals for each individual grid cell (for example, Fig. 1d), we used a basic *z* calculation in a *χ*^2^ framework:


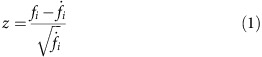


Where *f* was our observed rasterized species count of mimics for cell *i* and 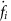 was the mean mimetic species count for the same grid cell from the randomized simulations.

### Phylogenetic tree estimation

For analyses conducted across all snakes, we used the time-calibrated squamate phylogeny from ref. 29 and pruned the tree to the monophyletic Serpentes clade. For analyses within the colubrid snake tribe Sonorini, we sequenced 6 nuclear (*c-mos*, *RAG1*, *AKAP-9*, *CILP*, *FSHR*, and *NTF-3*; primers from refs 51 and 52, sequences in Supplementary Data 6) and 2 mitochondrial loci (*cytb* and *ND4*; primers from ref. 52, sequences in Supplementary Data 6) for 60 tissue samples representing 21 species (Supplementary Data 5) for a total of 6,375 bp. Sequences for 11 additional species were taken from GenBank (for *cytb* and *c-mos*) or published sources (for *ND4* (refs 53, 54)). Forty-two of the tissue samples were population-level sampling within the *Sonora semiannulata* (Western Ground Snake) species complex, targeting populations for which we already had phenotypic data on colour pattern from the sampling of 1,760 snakes (collection details and individual population sample sizes given in ref. 31). Sanger sequencing was conducted using standard conditions and protocols.

We edited and aligned all sequences by locus in Geneious v6.1.8 using the Muscle algorithm after translating each sequence to manually check for erroneous amino acid stop codons indicating editing mistakes. We then constructed phylogenetic trees with the concatenated data set partitioned by gene in RaxML (ref. 55) using the GTRGAMMA model. We calculated nodal support for the best scoring ML tree by bootstrapping (1,000 replicates).

### Ancestral state reconstruction

To reconstruct colouration and infer the evolutionary history of mimicry, we first used a random local clock model^56^ (‘RLC') implemented in BEAST v1.8 (ref. 57) and analysed RBB coloration as a single character alignment along a fixed tree^29^ following the approach of ref. 58 by modifying the model of molecular evolution to reconstruct discrete character states. We chose this method due to its ability to accommodate rate heterogeneity among clades within large phylogenetic trees, which can cause erroneous character reconstructions when using traditional methods that all assume homogenous rates across clades (see ref. 58 for detailed argument and extensive model testing). We used similar parameter priors, starting values and linked distributions following the xml file in ref. 58, conservatively using a relatively flat prior on mean rate of trait evolution (0.001709; parsimony-inferred changes divided by tree length) to adequately explore both fast and slow rates of evolution. We performed three independent runs of 300 million generations each and confirmed convergence by comparison in Tracer^59^. All parameters had ESS values >100. From our posterior distributions of reconstructed characters, we used a ‘maximum credibility' approach implemented in the R package ‘BAMMtools'^60^ to accommodate uncertainty and choose the best supported configuration of gains and losses of mimicry.

Next, we analysed RBB coloration using discrete, time-reversible models of character evolution^61^ in a combination of maximum likelihood and Bayesian frameworks (‘ML'). Again to accommodate evolutionary rate heterogeneity, we developed a maximum likelihood approach for identifying the number and location of distinct evolutionary rate partitions across the snake phylogeny similar to the R package ‘MEDUSA'^62^ but that allowed for the modelling of discrete instead of continuous traits. After initially fitting a model with a single transition rate matrix to the full data set, we fit a series of nested character evolution models to the phylogeny using stepwise AIC. This approach assumes that the shifts to and from mimetic coloration anywhere on the phylogeny can be described by rate parameters characterizing origins (q01) and reversals (q10) of mimetic coloration. We then fit a derived model where one portion of the tree evolved under a second rate matrix, thus allowing a single subclade to have transition rates that are decoupled from those across the remainder of the phylogeny.

We split the phylogeny at all internal nodes with at least five descendant species and maximized the likelihood of the model with two rate matrices. We then compared model fit using AIC scores, where we treated the location of the rate shift as a free parameter. Thus, a model with two transition matrices has four rate parameters (q01-A, q10-A, q01-B and q10-B) plus a fifth parameter for the topological location of the rate shift. We rejected the simpler model in favour of the more complex model if ΔAIC was >2. We increased the complexity of the model incrementally, holding as fixed the location of the shift for the two-matrix model but attempting to add a third transition matrix (3-matrix model: 6 rate parameters+2 shift location parameters). We repeated the optimization and model selection procedure described above until further model complexity failed to improve the fit of the model to the data, identifying a total of nine distinct transition rate partitions across the tree (Supplementary Fig. 7b).

Given this set of transition rate partitions, we then estimated evolutionary rates for each partition in a Bayesian framework. We simulated posterior distributions of rate parameters for each partition using Markov chain Monte Carlo (MCMC). We used a hybrid maximum likelihood (location of partitions) and Bayesian (parameters on partitions) framework because transition rate estimates for several partitions with rare character state changes were associated with large confidence intervals. We chose a prior by using maximum likelihood to estimate the ‘whole tree' transition rate under a symmetric Markov model. We used an exponential distribution with a mean 10 × greater than this whole-tree rate as a prior on all transition rate parameters. This is a relatively flat distribution that allows substantially faster rates relative to the background rate while also minimizing extreme rates across the tree.

We reconstructed ancestral character states across the tree by sampling transition rate parameters from their joint posterior distribution and performing stochastic character mapping across the tree^63^, following previous implementations (for example, Phytools^64^). First, we sample a set of transition rate parameters from their joint posterior probability distribution. We then calculate conditional likelihoods down the tree using Felsenstein's pruning algorithm^65^. At the root of the tree, we obtain paired conditional likelihoods for our two states. We placed a prior of 0.05 on mimetic coloration at the root of the tree, because this state has an observed frequency of only 0.023 across the portion of the tips described by this rate partition, and because the equilibrium frequency of this state implied by the rates themselves is very low (for example, 1—q01/(q10+q01)=0.037). We then sampled a root state from the posterior at the root and then compute conditional probabilities at each successive descendant node and choose character states proportional to these probabilities. Finally, we simulate character histories on each branch conditional on the starting and ending node states, and the associated transition rate parameters. We performed 500 stochastic character maps to obtain ‘pseudo-histories' of the evolution of mimetic colouration. The frequency of the trait at any point in time was computed at 1,000 time points by counting all the branches at *t*_*i*_ that were assigned to each state. Because this is a stochastic character map, any given branch may include multiple character state changes; these were tracked explicitly.

To assess the robustness of our colour pattern reconstructions across methods, we also reconstructed ancestral states using a simple parsimony-based method of ancestral state estimation (which accommodates infinite rate heterogeneity across clades) implemented in the R package ‘phangorn'^66^ and compared the results to both the RLC and ML reconstructions. The results from all three methods were generally congruent (Supplementary Figs 4–6), although the RLC model did not perform as well as the other two methods in handling extreme rate heterogeneity among clades, as evidenced by (a) reconstruction of a few ancestral nodes as mimetic when none of the descendent tip taxa have RBB coloration (for example, the *Echinathera/Taeniophallus/Sordellina* clade in Dipsadines; Supplementary Fig. 5) and (b) a strong bias for inferring extra losses in large clades with the greatest variation in tip states, which was not seen in the ML or parsimony reconstructions.

Due to increased risk of erroneous inflation of transitions near the tree tips due to taxonomic uncertainty, the ‘number of transitions' to mimicry in New World colubrids that we report in the main text (‘at least 19') is a conservative estimate qualitatively inferred from the trait reconstructions and thus is much smaller than the average number of reconstructed origins across the entire tree (∼60; Supplementary Fig. 8), as a broad accommodation of uncertainty in the phylogenetic reconstruction of taxon relationships.

We repeated these analyses for the time-rooted (root calibrated to the *Sonora*-*Gyalopion* MRCA node date from ref. 19), species-level version of the Sonorini tree (Fig. 3c) but not for the population-level data (Fig. 3d), as intraspecific inference violates the key assumption of a strict branching process necessary for state reconstruction^67^.

### Geographic distribution of RBB gains and losses

We reconstructed the geographic history of RBB gain and loss across the snake phylogeny to address broad-scale latitudinal patterns in the frequency with which mimetic coloration is lost. We treated the latitudinal midpoint of each New World species as a continuous character and reconstructed the distribution of range midpoints across the phylogeny under a multi-rate Brownian motion process. We used BAMM v. 2.5 (ref. 68) to simulate a posterior distribution of geographic range states across all interior nodes in the phylogeny; the model explicitly allows the tempo and mode of geographic range evolution to vary among clades and thus affords some advantages relative to assuming a single fixed rate of range evolution. We then used stochastic character mapping under the best model for RBB coloration evolution (with eight shifts) to generate distributions of RBB gains and losses on individual branches across the phylogeny. For each observed gain and loss on a single branch in the stochastic character maps, we sampled an estimate of the latitudinal midpoint at that particular time and topological location using the posterior distribution of geographic node states simulated using BAMM. Because most RBB character state changes in these simulations occurred along branches and not at nodes, we linearly interpolated the geographic range state from the observed node states at the descendant and parent nodes. Thus, if a shift occurred on the middle of a particular branch, the geographic state assigned to the shift would be equal to the mean of the node states at the parent and descendant nodes. Letting *s*_x_ and *s*_y_ denote the geographic range states for ancestral and parent nodes, the interpolated state is given by the simple weighted average *δs*_x_+(1—*δ*)*s*_y_, where *δ* denotes the fractional time of occurrence of a shift alone a particular branch (for example, *δ*=0.5 corresponds to the middle of the branch; *δ*=0.1 denotes a state change occurring at 10% of the branch length between nodes *x* and *y*). We summarized the frequency distribution of RBB gains and losses with respect to latitude by applying kernel density estimation to vectors of inferred loss and gain locations. We treat this analysis provisionally, given the complexity of reconstructing geographic ranges. However, we observed a strong signal of phylogenetic conservatism in geographic range distributions. The variance in phylogenetic independent contrasts on latitudinal midpoint is much lower than expected under random permutations of tip states (*P*<0.001; observed mean: 8.9; simulated mean: 60.6; *z* score: −6.664), indicating that taxa with similar latitudinal midpoints are highly clustered on the tree. Hence, we interpret this analysis as reflecting broad scale trends (for example, tropics versus temperate zone) in the frequency of gains and losses.

## Additional information

**Accession codes:** Geographic range data for New World snakes are archived at Dryad (http://dx.doi.org/10.5061/dryad.qk300) and nucleotide sequences for all newly acquired Sonorini species are deposited in the Genbank nucleotide database under accession codes KU859402 to KU859865.

**How to cite this article:** Davis Rabosky, A. R. *et al*. Coral snakes predict the evolution of mimicry across new world snakes. *Nat. Commun.* 7:11484 doi: 10.1038/ncomms11484 (2016).

## Supplementary Material

Supplementary Information Supplementary Figures 1-9, Supplementary Note 1 and Supplementary References

Supplementary Data 1 Phenotypic classifications for all coral snakes, presumed mimics, and Old World snakes with red-black banded (RBB) coloration (n = 255 species). Phenotype codes follow ^29^(see for full code descriptions and pictorial representations). NW = New World, OW = Old World, N = Not present, Y = Present. Reference numbers refer to Supplementary References.

Supplementary Data 2 Latitude-longitude grid cell data for species richness and abundance counts at 0.5° resolution. Column codes: x = longitude, y = latitude, onland = presence on land (0 = No, 1 = Yes).

Supplementary Data 3 Latitude-longitude grid cell data for species richness and abundance counts at 2° resolution. Column codes: x = longitude, y = latitude, onland = presence on land (0 = No, 1 = Yes).

Supplementary Data 4 Transition rate parameters for the evolution of mimetic coloration in New World snakes for the best-fit model of trait evolution. Taxon 1 and taxon 2 span the node at which the transition rate shift was inferred to occur. q_01_ and q_10_ denote instantaneous rates of gain and loss of RBB coloration (means of the posterior rate distributions simulated using MCMC). These are numerical rates corresponding to Supplementary Figure 7.

Supplementary Data 5 Specimen and GenBank information for all sequenced Sonorini (*n* = 71 individuals). B = presence of crosswise black bands, R = presence of red bands or full dorsum coloration, - = coloration absent on any individuals, Y= yes, N = no.

Supplementary Data 6 Primer sequences.
