# Supplementary material for: Coral snakes predict the evolution of mimicry across New World snakes
Source: Nat Commun. 2016 May 5;7:11484. doi: 10.1038/ncomms11484 (PMC4858746; doi:10.1038/ncomms11484)
Supplement: Supplementary Information — Supplementary Figures 1-9, Supplementary Note 1 and Supplementary References [file ncomms11484-s1.pdf]

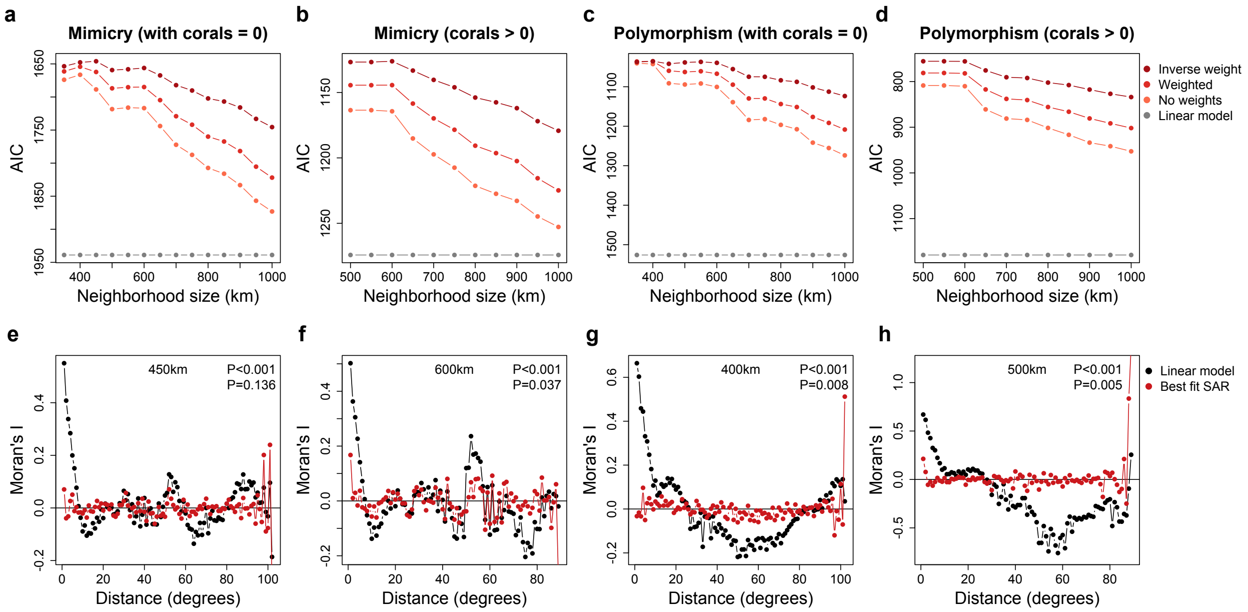

Supplementary Figure 1. AIC scores and Moran's I values for spatial autoregressive (SAR) models. a-d, AIC values for increasing neighbourhood sizes and weighting regimes show that inverse weighting by distance performed the best across analyses and that all SAR models were a much better fit than simple linear models. The dependent variable and whether grid cells with no coral snakes present were included is given in panel titles and correspond to E-H below. **e-h,** Moran's I values for linear and SAR models show that the best fit SAR models removed most of the spatial autocorrelation present in the data. Best fit neighbourhood size and *P* values (full model above, coral snake effect below) are given at the top of each panel.

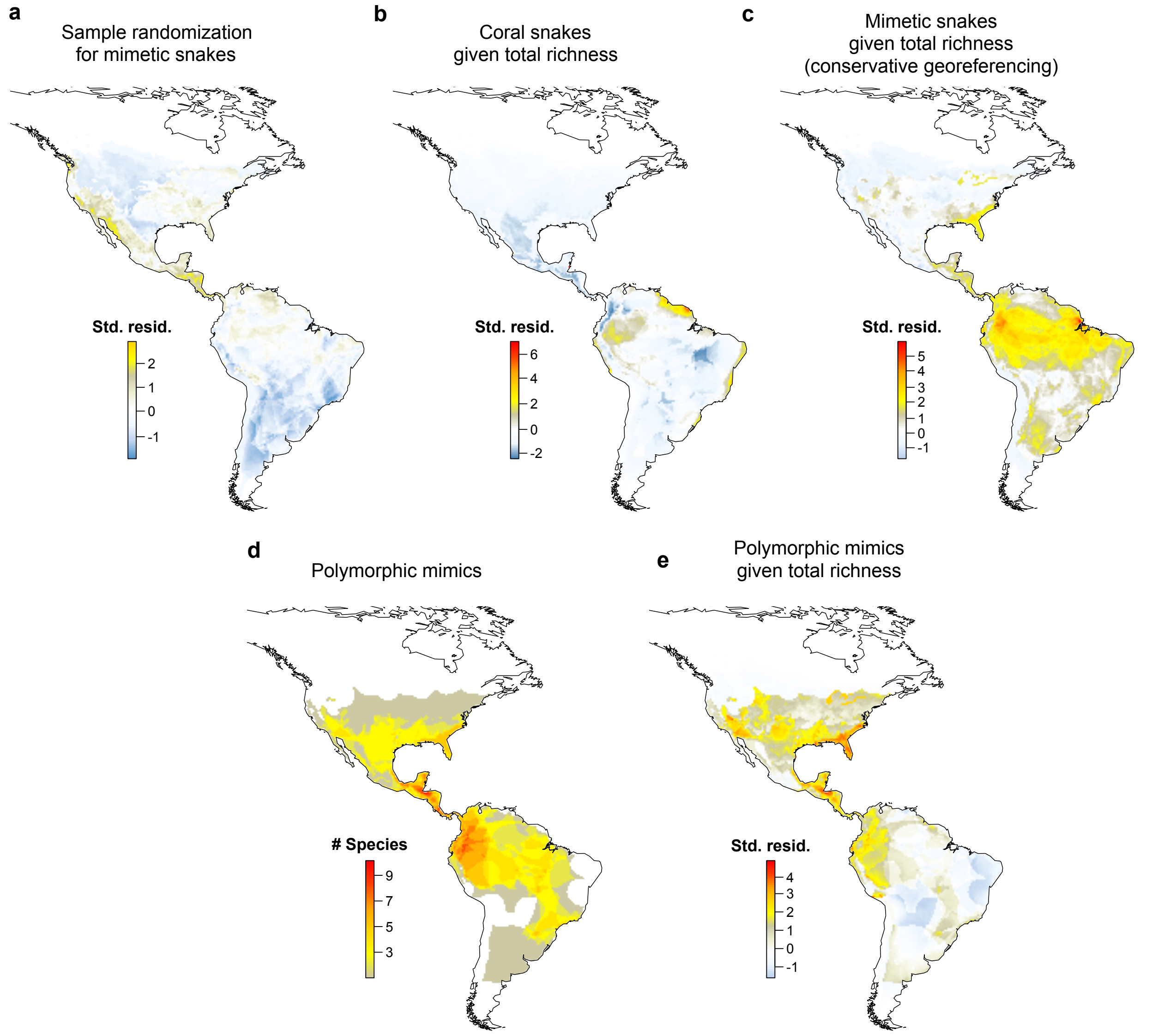

Supplementary Figure 2. Supplementary spatial plots for mimicry and polymorphism. a, Standardised residuals of mimetic snakes for a single randomisation compared to the simulated means for each grid cell confirm that the null distributions perform well (nearly all grid cell values between ±2 s.d. and minimal spatial pattern to outlier cells). Yellow and warmer colours denote significant positive deviation in all panels. **b,** Standardised residuals of observed coral snakes to simulated means confirm no complex interaction effect between coral snake and overall species richness. **c,** Standardised residuals of observed mimicry data under a conservative georeferencing regime are qualitatively similar to results with non-georeferenced records placed randomly within collection state (Fig. 1d). **d,** Observed species richness distribution of mimetic species with colour polymorphism (*n* = 46 species). **e,** Standardised residuals of polymorphic mimics show a more complicated geographic pattern in which polymorphism is high both in areas with many coral snake species (western Amazon, Central America) as well as in allopatry to coral snakes (western United States).

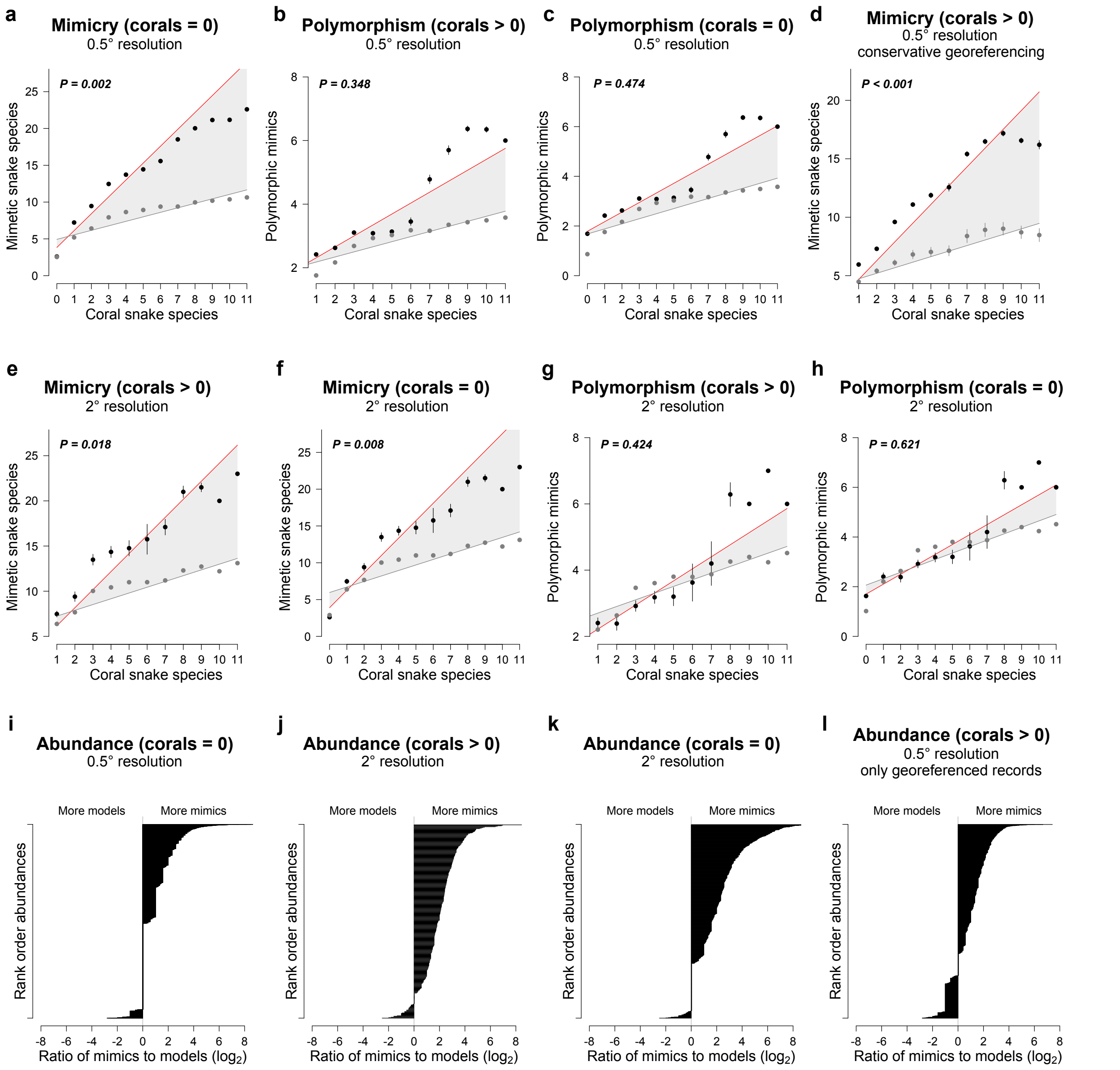

Supplementary Figure 3. Supplementary regression and abundance plots.
a, Regression models including grid cells containing no coral snakes show the same result as regressions with only cells where coral snakes are present (Fig. 2a). For all regression plots, black points are observed data and grey dots are expected values from simulation. **b,** Weak but nonsignificant positive correlation between the number of polymorphic mimics and number of coral snakes, excluding cells with no coral snakes. **c,** Weak but nonsignificant positive correlation between polymorphism and coral snakes when including cells with no coral snakes. **d,** More conservative placement of non-georeferenced occurrences shows the same result as our original georeferencing pipeline (Fig. 2a). **e-h,** Repeated richness analyses at a 2° grid cell resolution. **i,** Abundance plot including grid cells with no coral snakes show increased ratios of 0 but do not change the overall conclusion that mimetic snakes are often more abundant than coral snakes. **j-k,** Repeated abundance analyses at a 2° grid cell resolution. **l,** Using only georeferenced occurrence records show the same pattern as the full record set (Fig. 2b).

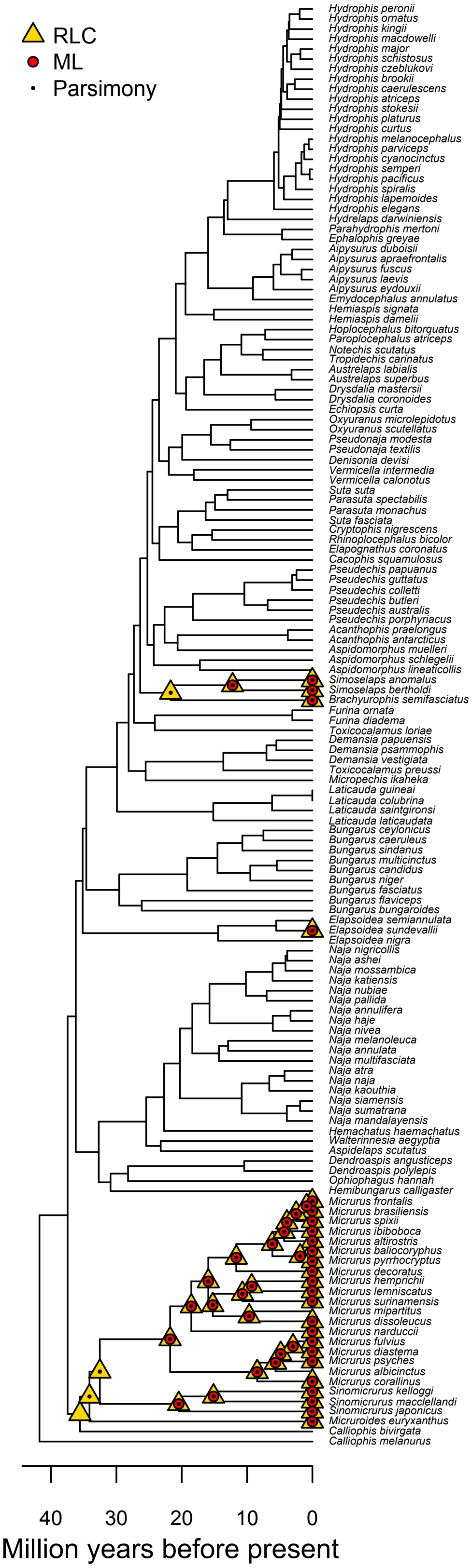

Supplementary Figure 4. Red-black banded (RBB) warning coloration in Elapidae. Character reconstructions for the snake family Elapidae using three different methods show multiple independent origins of RBB coloration. Marked nodes are those reconstructed as red-black banded.

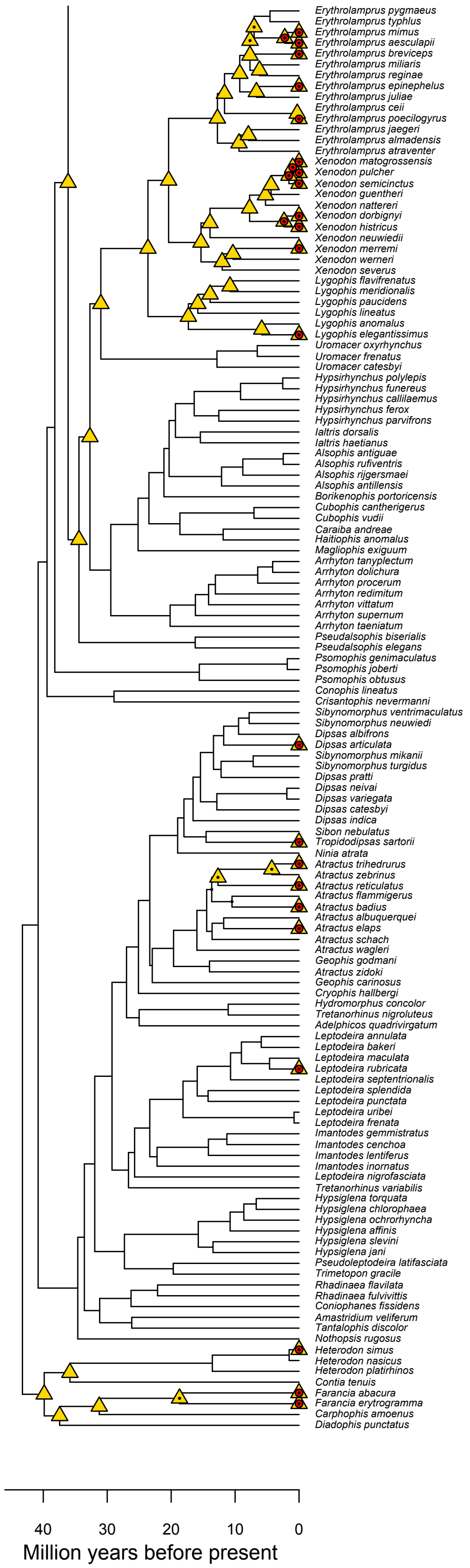

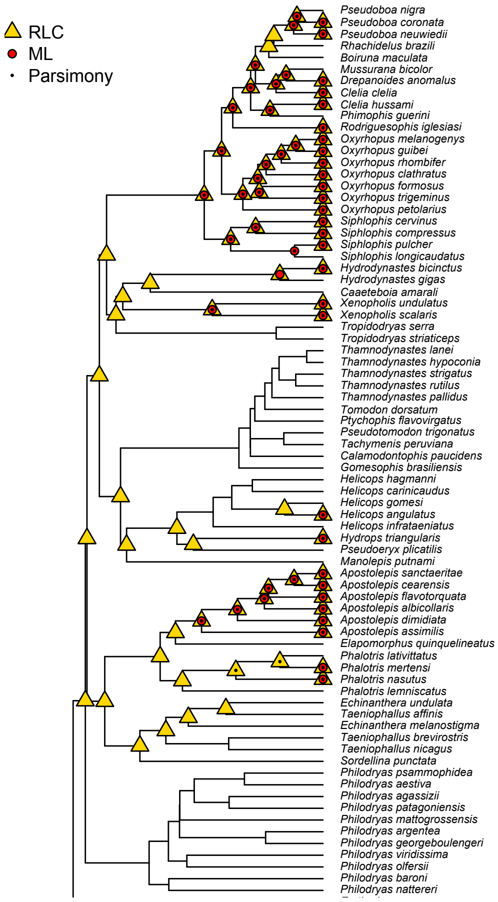

Supplementary Figure 5. Red-black banded (RBB) mimetic coloration in Dipsadines. Character reconstructions for the subfamily Dipsadinae (family Colubridae) using three different methods show multiple independent origins of RBB coloration. Marked nodes are those reconstructed as red-black banded. Tree fragment on the right follows from the top of the tree fragment on the left.

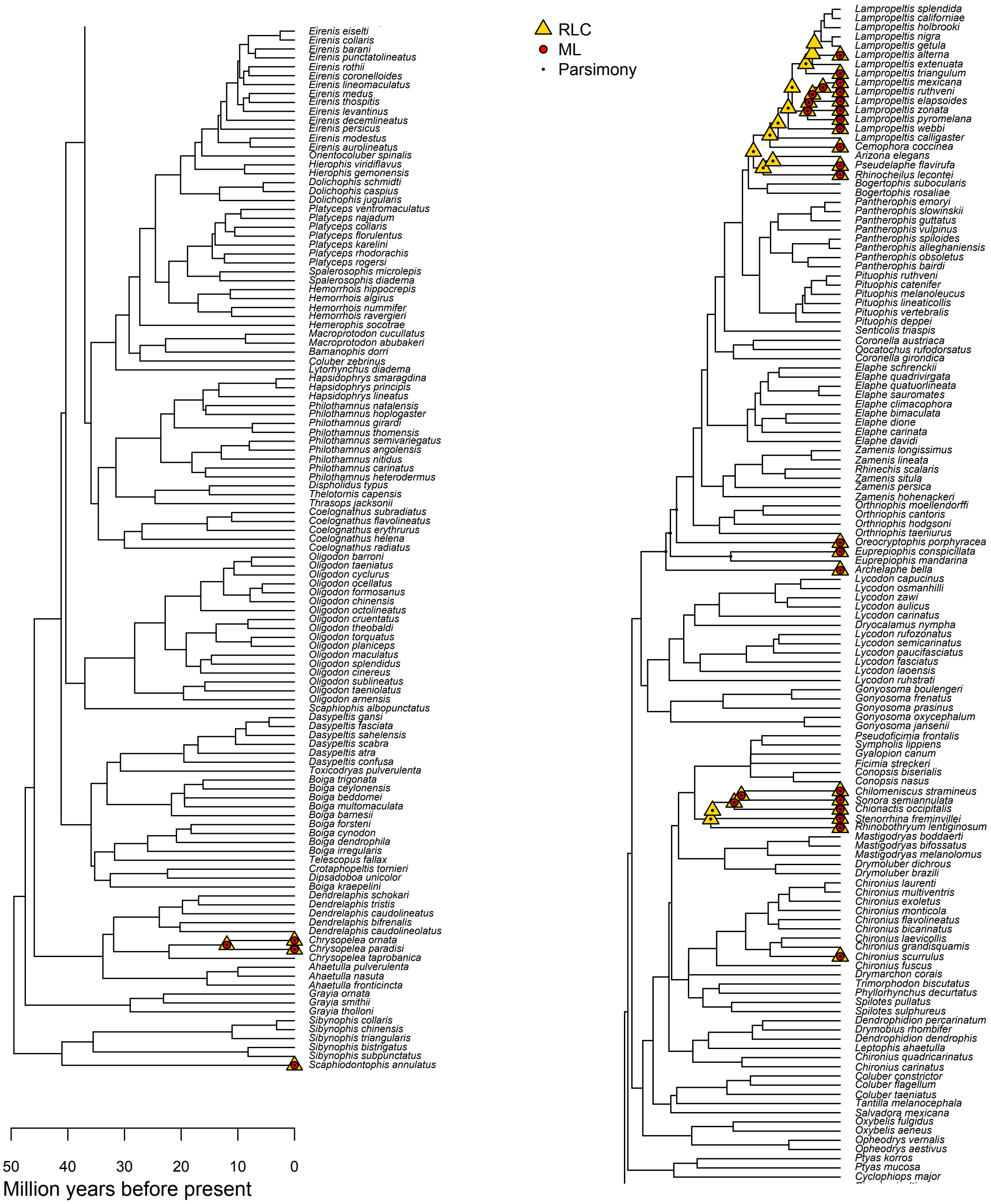

Supplementary Figure 6. Red-black banded (RBB) mimetic coloration in Colubrines. Character reconstructions for the subfamily Colubrinae (family Colubridae) using three different methods show multiple independent origins of RBB coloration. Marked nodes are those reconstructed as red-black banded. Tree fragment on the right follows from the top of the tree fragment on the left.

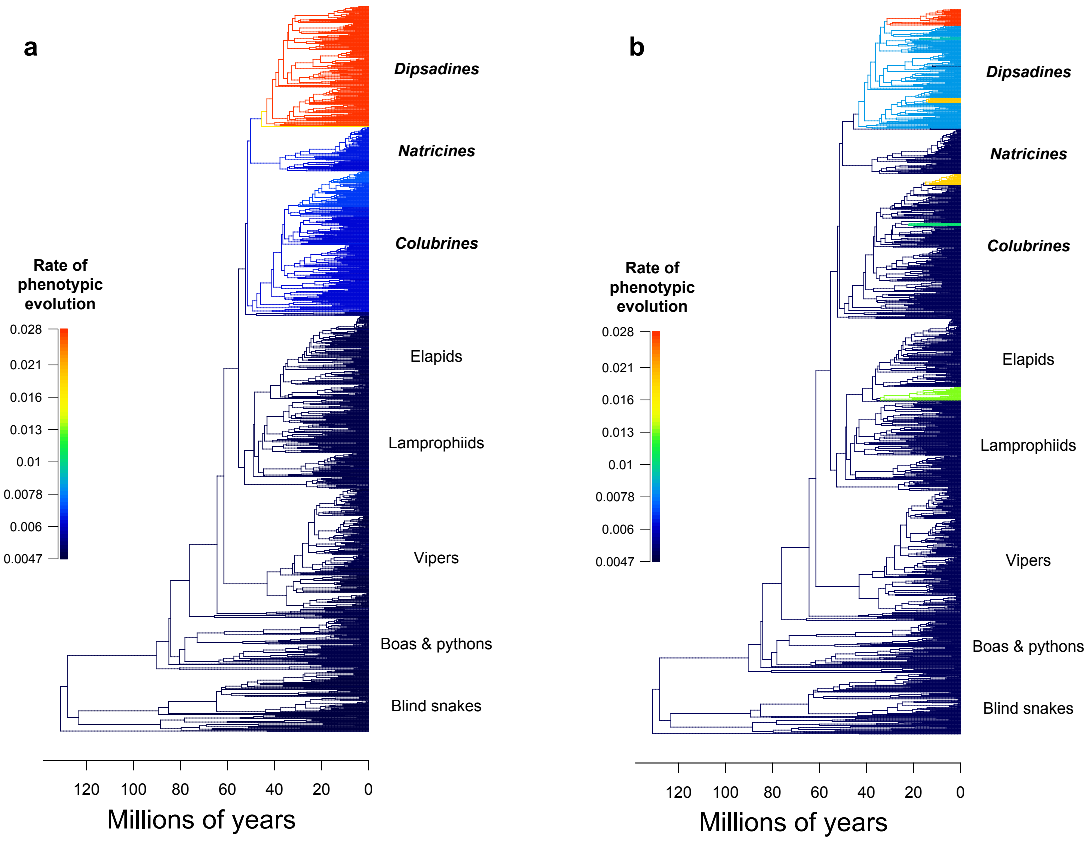

Supplementary Figure 7. Rates of phenotypic evolution. a, Phylogenetic tree with branches coloured by rate of colour pattern evolution (number of changes per million years) from the random local clock (RLC) model show a much higher rate in the subfamily Dipsadinae. Units correspond to number of changes per million years. **b,** Rate estimation under the maximum likelihood (ML) model shows greater rate heterogeneity than the RLC model.

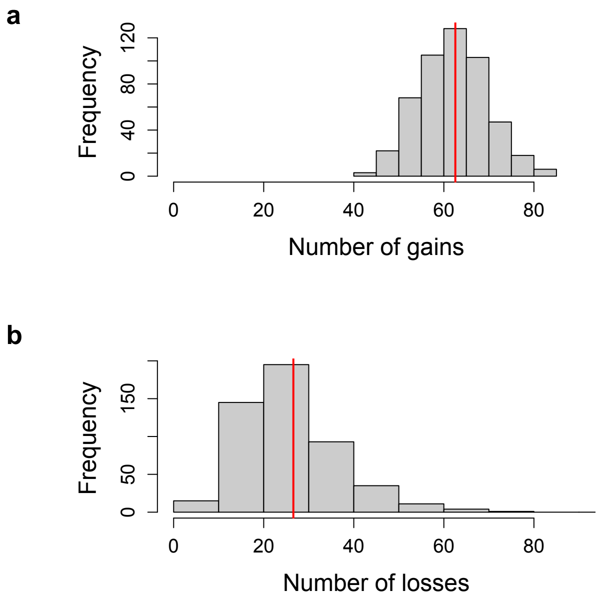

Supplementary Figure 8. Losses and gains of red-black banded coloration. Frequency histograms of 500 character reconstruction permutations using the maximum likelihood + stochastic character mapping method recover more than twice as many gains (**a**) of RBB coloration as losses (**b**), although losses of mimicry are still quite common. Means are indicated by vertical red lines.

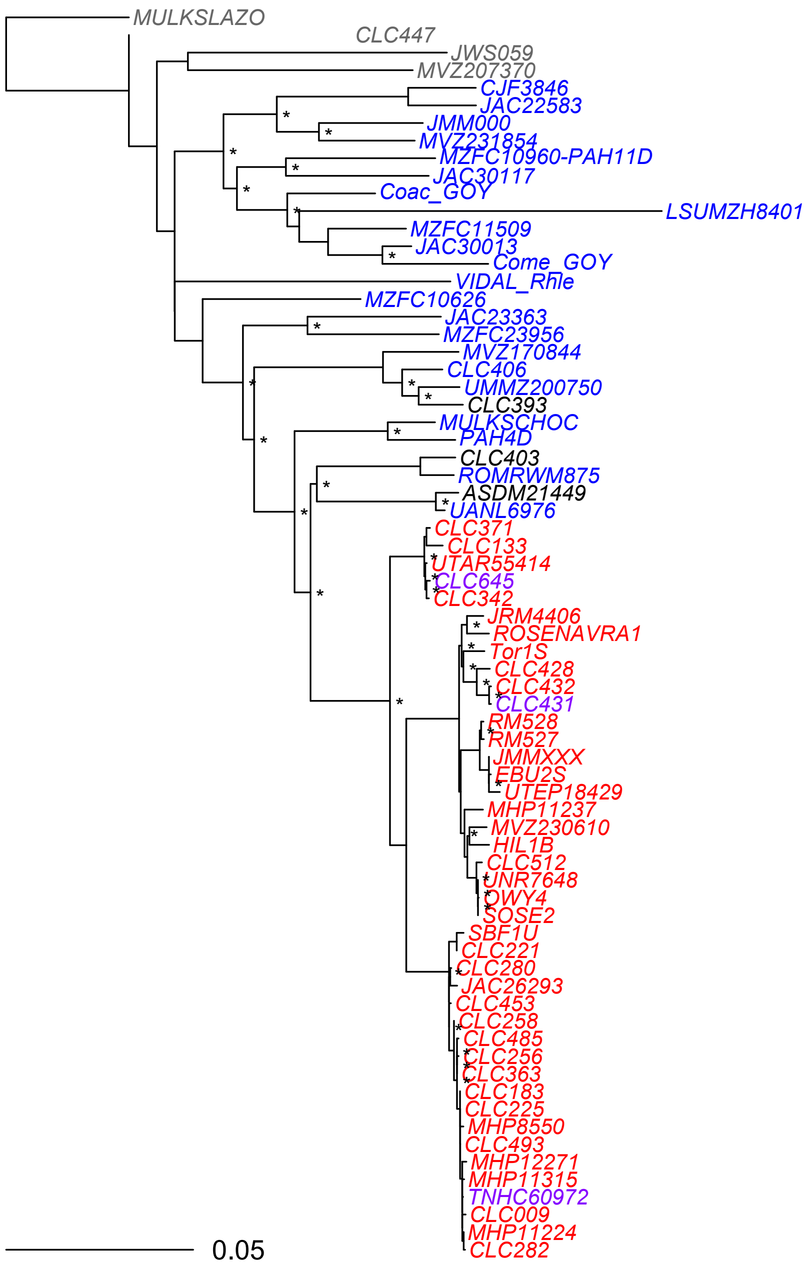

Supplementary Figure 9. Phylogeny of the Sonorini from 6 nuclear and 2 mitochondrial genes. Maximum likelihood reconstruction of the phylogenetic relationships of 22 species within the snake tribe Sonorini, including population-level sampling within the *Sonora semiannulata* species complex. Taxon labels and bootstrapped node support values are provided for comparison to Fig. 3c and Fig. 3d. Blue taxa were included in the species-level phylogeny in Fig. 3c, red taxa in Fig. 3d, and purple taxa in both. Grey taxa are outgroups. Corresponding specimen data and GenBank numbers are in Supplementary Table 5. Asterisks show node support > 50.

Supplementary Note 1

It should be noted that although our spatial dataset is only ~83% complete due to lack of recorded observations in museums, we do not expect these missing species to fundamentally alter our conclusions. The species missing from the richness dataset are generally endemics with very small ranges (as widespread species are most likely to be represented in collections) and the species missing from the abundance dataset are generally quite rare. Thus, the numerical impact of missing data in our analyses is expected to be minimal, as both species richness and abundance grid cell totals would only be negligibly increased by the inclusion of sparsely distributed data given the large overall size of both datasets.

We note that our main temporal tests do not explicitly include full ancestral range reconstructions to validate "ensured historical sympatry" between coral snakes and their mimics. Rather, our temporal correlation simply tests for the *earliest opportunity* in which these clades could have been in sympatry in the New World. Because the fossil record for New World snakes is patchy and sparse (although coral snake vertebrae are known from the Miocene of Nebraska, USA, so coral snakes were historically distributed further north than their current distribution), we originally assumed nothing about the historical distribution of coral snakes other than the timing of their earliest possible arrival to the New World from the molecular split with Asian coral snakes. Although the absolute values of all divergences times may be skewed, that bias should not systematically affect the relative position of these values to each other, which is the metric of interest in our assessment of the timing of RBB origin in coral snakes versus New World colubrids. Although we then reconstructed latitudinal midpoints of geographic ranges as continuous variables across the tree to generate a profile of the geographic distribution of

coloration changes, we note that these are not "full range" constructions - they are are only latitudinal midpoints. Thus, we urge that this analysis should be interpreted conservatively. Because there is considerable geographic conservatism to clade distributions (*e.g.*, there is a large Dipsadine clade that does not expand out of the tropics), we think that this analysis is informative about the relative rate of gain and loss in the tropics and near the margins of the present-day coral snake distribution. Beyond this coarse generalisation, though, we encourage caution in making anything more significant out of this finding.

Additionally, although many taxa are missing from the tree due to lack of DNA sequence information, the overall rarity of the RBB coloration suggests that the future addition of missing taxa will mainly serve to confirm or increase - rather than decrease - the number of transitions recovered here. Transitions could be pushed further back in time by the fortuitous, balanced placement of missing taxa specifically spanning transition points, but numbers of reconstructed origins are far more likely to increase rather than decrease without any changes to the timing of the origin of mimicry within clades (as we found by adding taxa to the Sonorini clade).

We also note that there are two New World mimetic species each on single, long branches (Colubridae: Neckband snakes, *Scaphiodontophis annulatus*; and Aniliidae: South American pipe snakes, *Anilius scytale*) for which we have no information to reliably date the RBB origin. The branch lengths for these species are ~40 and ~80my respectively, and our stochastic character mapping simulations produce essentially uniform distributions of the timing of the RBB origin across each branch. Thus, we do not consider either taxon to be an exception to our finding that RBB coloration in non-venomous snakes arose after the earliest possible arrival of coral snakes

in the New World, but rather that due to their unique topology, we have no information about the timing in these two lineages to reconstruct these origins with any confidence.

Supplementary References

1. Savage J. S., Slowinski J. B. The colouration of the venomous coral snakes (family Elapidae) and their mimics (families Aniliidae and Colubridae). *Biol. J. Linnean Soc.* **45**, 235-254 (1992).

2. Nogueira, C., Barbo, F. E. & Ferrarezzi, H. Redescription of *Apostolepis albicollaris* Lema, 2002, with a key for the species groups of the genus *Apostolepis* (Serpentes: Dipsadidae: Elapomorphini). *South Am. J. Herpetol.* **7**, 213-225 (2012).

3. Ferrarezzi, H., Barbo, F. E. & Albuquerque, C. E. Phylogenetic relationships of a new species of *Apostolepis* from Brazilian Cerrado with notes on the *assimilis* group (Serpentes: Colubridae: Xenodontinae: Elapomorphini). *Pap. Avulsos de Zool.* **45**, 215-229 (2005).

4. Harvey, M. B., A, L. G. & Scrocchi, G. J. New species of *Apostolepis* (Squamata: Colubridae) from the Gran Chaco in Southern Bolivia. *Copeia* **2001**, 501-507 (2001).

5. Das, I. *A field guide to the reptiles of South-East Asia*. (Bloomsbury Publishing, 2015).

6. Alexander, G. & Marais, J. *A guide to the reptiles of southern Africa*. (Struik Pub, 2007).

7. Campbell J. A. & Lamar W. W. *The Venomous Reptiles of the Western Hemisphere*. Cornell University Press (2004).

8. BSCIT, U. o. C., Berkeley. *CalPhotos: A database of photos of plants, animals, habitats and other natural history subjects*, <http://calphotos.berkeley.edu. > (2015).

9. Passos, P. *et al.* A new species of *Atractus* (Serpentes: Dipsadidae) from Serra do Cipó, Espinhaço Range, Southeastern Brazil, with proposition of a new species group to the genus. *Pap. Avulsos de Zool.* **53**, 75-85 (2013).

10. Myers, C. W. & Schargel, W. E. Morphological extremes – two new snakes of the genus *Atractus* from northwestern South America (Colubridae: Dipsadinae). *Am. Mus. Novit.* **3532**, 1-13 (2006).

11. Orlov, N. L., Ryabov, S. A. & Nguyen, T. T. On the taxonomy and the distribution of snakes of the genus *Azemiops* Boulenger, 1888: Description of a new species. *Russ. J. Herpetol.* **20**, 110-128 (2013).

12. O'Shea, M. *Boas and Pythons of the World*. (New Holland Publishers, 2007).

13. Wilson, S. & Swan, G. *Complete guide to reptiles of Australia*. 4th edition edn, (New Holland Publishers, 2013).

14. Uetz, P. & Hošek, J. *EMBL/EBI Reptile Database* <http://www.reptile-database.org>. (1996-present).

15. Spawls, S., Howell, K., Drewes, R. C. & Ashe, J. *Field guide to the reptiles of East Africa: Kenya, Tanzania, Uganda, Rwanda and Burundi*. (A&C Black, Bloomsbury Publishing, 2004).

16. Savage, J. M. *The Amphibians and Reptiles of Costa Rica: A Herpetofauna Between Two Continents, Between Two Seas*. (University of Chicago Press, 2002).

17. Wilson, L. D., McCranie, J. R. & Williams, K. L. A new species of *Geophis* of the *sieboldi* group (Reptilia: Squamata: Colubridae) from northern Honduras. *Proc. Biol. Soc. Wash.* **111**, 410-417 (1998).

18. De La Torre-Lacano, M. A., Aguirre-León, G. & López-Luna, M. A. Coralillos verdaderos (Serpentes: Elapidae) y coralillos falsos (Serpentes: Colubridae) de Veracruz, México. *Acta Zool. Mex.* **22**, 11-22 (2006).

19. Townsend, J. H. Morphological variation in *Geophis nephodrymus* (Squamata: Colubridae), with comments on conservation of *Geophis* in eastern nuclear Central America. *Herpetologica* **65**, 292-302 (2009).

20. Pavón-Vázquez, C. J., García-Vázquez, U. O., Blancas-Hernández, J. C. & de Oca, A. N.-M. A new species of the *Geophis sieboldi* group (Squamata: Colubridae) exhibiting color pattern polymorphism from Guerrero, Mexico. *Herpetologica* **67**, 332-343 (2011).

21. Pyron, R. A. & Burbrink, F. Body size as a primary determinant of ecomorphological diversification and the evolution of mimicry in the lampropeltinine snakes (Serpentes: Colubridae). *J. Evol. Biol.* **22**, 2057-2067 (2009).

22. Bryson Jr, R. W., Dixon, J. R. & Lazcano, D. New species of Lampropeltis (Serpentes: Colubridae) from the Sierra Madre Occidental, Mexico. *J. Herpetol.* **39**, 207-214 (2005).

23. Dixon, J. R. A review of *Liophis anomalus* and *Liophis elegantissimus*, and the description of a new species (Serpentes: Colubridae). *Copeia*, 565-573 (1985).

24. Roze J. A. *Coral snakes of the Americas: Biology, identification, and venoms*. Krieger Publishing Company (1996).

25. Campbell, J. A. & Lamar, W. W. *The Venomous Reptiles of Latin America*. (1989).

26. da Silva Jr, N. J. & Sites Jr, J. W. Revision of the *Micrurus frontalis* complex (Serpentes: Elapidae). *Herpetol. Monogr.* **13**, 142-194 (1999).

27. Renjifo, J. M. & Lundberg, M. Una especie nueva de serpiente coral (Elapidae, Micrurus), de la region de Urra, municipio de Tierra Alta, Cordoba, noroccidente de Colombia. *Revista Acad. Colomb. Ci. Exact.* **27**, 141-144 (2003).

28. Carvalho, C. M. d. Descrição de uma nova espécie de *Micrurus* do Estado de Roraima, Brasil (Serpentes, Elapidae). *Pap. Avulsos de Zool.* **42**, 183-192 (2002).

29. Campbell, J. A. A new species of venomous coral snake (Serpentes: Elapidae) from high desert in Puebla, Mexico. *Proc. Biol. Soc. Wash.* **113**, 291-297 (2000).

30. Pires, M. G., Da Silva Jr, N. J., Feitosa, D. T., Prudente, A. & Zaher, H. A new species of triadal coral snake of the genus *Micrurus* Wagler, 1824 (Serpentes: Elapidae) from northeastern Brazil. *Zootaxa*, 569-584 (2014).

31. Lamar, W. W. A new species of slender coralsnake from Colombia, and its clinal an ontogenetic variation (Serpentes, Elapidae: *Leptomicrurus*). *Rev. Biol. Trop.* **51**, 805-810 (2003).

32. Di-Bernardo, M., Borges-Martins, M. & da Silva Jr, N. J. A new species of coralsnake (*Micrurus*: Elapidae) from southern Brazil. *Zootaxa* **1447**, 1-26 (2007).

33. Lavin-Murcio, P. A. & Dixon, J. R. A new species of coral snake (Serpentes, Elapidae) from the Sierra de Tamaulipas, Mexico. *Phyllomedusa* **3**, 3-7 (2004).

34. Orlov, N. L. Cover Image. *Russ. J. Herpetol.* **17** (2010).

35. Bernardo, P. H., MacHado, F. A., Murphy, R. W. & Zaher, H. Redescription and morphological variation of *Oxyrhopus clathratus* Duméril, Bibron and Duméril, 1854 (Serpentes: Dipsadidae: Xenodontinae). *South Am. J. Herpetol.* **7**, 134-148 (2012).

36. Zaher, H. & Caramaschi, U. Synonymisation of *Oxyrhopus venezuelanus* Shreve, 1947, with *Oxyrhopus doliatus* Duméril, Bibron & Duméril, 1854, and revalidation of *Oxyrhopus erdisii* (Barbour, 1913)(Serpentes, Colubridae). *Dumerilia* **4**, 113-122 (2000).

37. Vriesendorp, C., Rivera Chávez, L., Moskovits, D. & Shopland, J. Perú: Megantoni., (The Field Museum, Chicago, Illinois, 2004).

38. Lynch, J. D. Snakes of the genus *Oxyrhopus* (Colubridae: Squamata) in Colombia: taxonomy and geographic variation. *Pap. Avulsos de Zool.* **49**, 319-337 (2009).

39. Moura, M. R., Costa, H. C. & Pirani, R. M. Rediscovery of *Phalotris concolor* (Serpentes: Dipsadidae: Elapomorphini). *Zoologia (Curitiba)* **30**, 430-436 (2013).

40. Hamdan, B., da Silva Jr., N. J., Silva, H. L. R., Cintra, C. E. D. & De Lema, T. Redescription of *Phalotris labiomaculatus* (Serpentes, Dipsadidae, Elapomorphini), with notes on the taxonomic boundaries within the *nasutus* group. *Zootaxa* **3693**, 182-188 (2013).

41. De Lema, T., D'Agostini, F. M. & Cappellari, L. H. Nova espécie de *Phalotris*, redescrição de *P. tricolor* e osteologia craniana (Serpentes, Elapomorphinae). *Iheringia, Sér. Zool.* **95**, 65-78 (2005).

42. Rovero, F. *et al.* Targeted vertebrate surveys enhance the faunal importance and improve explanatory models within the Eastern Arc Mountains of Kenya and Tanzania. *Divers. Distrib.* **20**, 1438-1449 (2014).

43. Zaher, H., Oliveira, M. E. & Franco, F. L. A new, brightly colored species of *Pseudoboa* Schneider, 1801 from the Amazon Basin (Serpentes, Xenodontinae). *Zootaxa* **1674**, 27-37 (2008).

44. Grazziotin, F. G. *et al.* Molecular phylogeny of the New World Dipsadidae (Serpentes: Colubroidea): a reappraisal. *Cladistics* **28**, 437-459 (2012).

45. Sheehy, C. M., Yánez-Muñoz, M. H., Valencia, J. H. & Smith, E. N. A new species of *Siphlophis* (Serpentes: Dipsadidae: Xenodontinae) from the eastern Andean slopes of Ecuador. *South Am. J. Herpetol.* **9**, 30-45 (2014).

46. Costa, H. C., São-Pedro, V. & Feio, R. N. A new record of the poorly known Amazonian snake *Siphlophis worontzowi*. *Herpetol. Notes* **3**, 97-100 (2010).

47. Cox C. L., *et al.* Molecular systematics of the genus *Sonora* (Squamata: Colubridae) in central and western Mexico. *Systematics and Biodiversity*, (2012).

48. Torres-Carvajal, O., Salazar-Valenzuela, D. & Merino-Viteri, A. *ReptiliaWebEcuador. Versión 2014.0*, <http://zoologia.puce.edu.ec/Vertebrados/reptiles/reptilesEcuador>, (2014).
